# A Quadrupolar Bis‐Triarylborane Chromophore as a Fluorimetric and Chirooptic Probe for Simultaneous and Selective Sensing of DNA, RNA and Proteins

**DOI:** 10.1002/chem.201903936

**Published:** 2020-01-22

**Authors:** Željka Ban, Stefanie Griesbeck, Sanja Tomić, Jörn Nitsch, Todd B. Marder, Ivo Piantanida

**Affiliations:** ^1^ Division of Organic Chemistry and Biochemistry Ruder Boškovic Institute Zagreb Croatia; ^2^ Institut für Anorganische Chemie and Institute for Sustainable Chemistry & Catalysis with Boron Julius-Maximilians-Universität Würzburg 97074 Würzburg Germany

**Keywords:** boranes, circular dichroism, fluorescent probes, luminescence, sensors

## Abstract

A water‐soluble tetracationic quadrupolar bis‐triarylborane chromophore showed strong binding to ds‐DNA, ds‐RNA, ss‐RNA, as well as to the naturally most abundant protein, BSA. The novel dye can distinguish between DNA/RNA and BSA by fluorescence emission separated by Δ$\tilde{\nu }$ =3600 cm^−1^, allowing for the simultaneous quantification of DNA/RNA and protein (BSA) in a mixture. The applicability of such fluorimetric differentiation in vitro was demonstrated, strongly supporting a protein‐like target as a dominant binding site of **1** in cells. Moreover, our dye also bound strongly to ss‐RNA, with the unusual rod‐like structure of the dye, decorated by four positive charges at its termini and having a hydrophobic core, acting as a spindle for wrapping A, C and U ss‐RNAs, but not poly G, the latter preserving its secondary structure. To the best of our knowledge, such unmatched, multifaceted binding activity of a small molecule toward DNA, RNA, and proteins and the selectivity of its fluorimetric and chirooptic response makes the quadrupolar bis‐triarylborane a novel chromophore/fluorophore moiety for biochemical applications.

## Graphical abstract

**Boron finds DNA, RNA and BSA**: A water‐soluble tetracationic quadrupolar bis‐triarylborane chromophore as ratiometric sensor for DNA/RNA and protein (BSA) is presented. This novel dye can distinguish between DNA/RNA and BSA by a characteristic fluorescence emission.

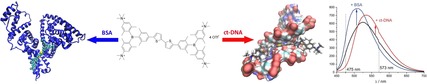

## Introduction

Low‐molecular‐weight fluorescent probes are frequently used tools in most molecular biology or biochemistry experiments today, as well as in many medical diagnostic tests.^1^ In order to avoid preparation of fluorescently labelled secondary monoclonal antibodies specific for some organelle, and therefore to simplify and accelerate experiments, non‐covalently binding molecular sensors that readily recognize and distinguish between different types of biomolecules are of huge interest.^2^ However, the small size of a molecule presents a significant challenge in the design of selective probes, particularly in well‐explored fluorophore families. Thus, development of a novel structural motif represents a valuable contribution. One of particular interest would be a small‐molecule probe which binds to several different biomacromolecules (e.g. DNA and protein) giving a different spectroscopic response for each type of biomolecule. That would allow for monitoring the cell life processes related to biomacromolecule targets and their mutual relations by only one dye instead of several.

We recently reported the water‐soluble and stable cationic bis‐triarylborane derivative **1** as a new fluorophore applicable for one‐ and two‐photon excited fluorescence imaging in cells.^3^ Given that only a few examples of water‐soluble triarylboranes have been reported to date,^4^, ^5^, ^6^, ^7^, ^8^, ^9^, ^10^, ^11^, ^12^, ^13^ such borane‐based chromophores could be considered to be novel fluorophores for biochemical applications; they are already widely used in nonlinear optical materials, organic electronics, organic light‐emitting diodes or anion sensors.^14^, ^15^, ^16^, ^17^, ^18^, ^19^, ^20^, ^21^, ^22^ However, fluorescence of **1** only enabled intracellular localisation of a dye, but did not allow for the determination of a targeted biomacromolecule (for example, protein or DNA or RNA). The question arose as to whether it is possible, by detailed analysis of the dye's spectrophotometric response in a cell, to determine the type of targeted biomacromolecule, which would help to determine the binding site and suggest further dye applications and developments.

**Scheme 1:**
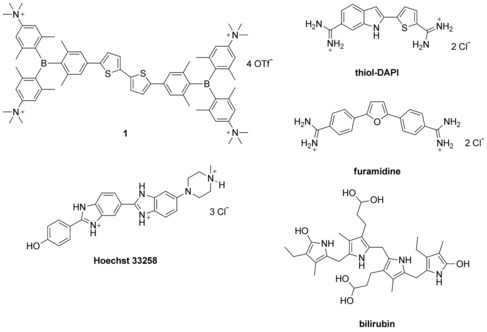
Schematic representations of our tetracationic bis‐triarylborane **1** and known DNA minor‐groove binders DAPI‐thiol‐analogue,^24^ furamidine,^25^ Hoechst 33258,^26^ and known HSA/BSA ligand bilirubin.^**[**33]^

Such elongated, rod‐like molecules, bearing four positive charges at the long axis termini, in part resemble bis‐amidine derivatives of five‐membered heterocyclic systems studied in detail by Wilson and Boykin.^23^, ^24^ Over the last decade we have explored in detail a large series of amidine‐substituted derivatives, combining structures of DAPI (4’,6‐diamidino‐2‐phenylindole), furamidine^25^ and Hoechst 33258^26^ (Scheme 1), whereby we systematically varied the molecular length, volume and rigidity.^27^, ^28^, ^29^ In particular, thiophene analogues showed intriguing DNA and RNA binding properties including, for example, stronger stabilisation of ds‐DNA with respect to other furanyl‐analogues^**[**27**]**^ and, in some cases, showed promising antiproliferative effects.^30^, ^31^ Our results showed that a rather fine interplay between planarity of the aromatic system, its flexibility, distribution of positive charge and choice of central heterocyclic system can control not only the DNA/RNA binding mode (intercalation, minor groove binding, DNA/RNA groove aggregation) but, in thiophene analogues, could result in a switch of intracellular target, for example, from DNA to tubulin.^30^

Therefore, in particular, the terminal dicationic triarylborane groups in novel molecule **1** could be considered as new positively charged termini of exceptionally large volume with respect to all of our previously studied positive terminal groups. Moreover, the triarylboranes are also fluorophores, thus able to sense interactions with DNA, RNA or protein target directly.

The pronounced hydrophobicity of **1** in the central dithiophene core (Scheme 1), prompted us to study its interactions with serum albumin (BSA), a known physiological transporter of a variety of heterocyclic molecules^32^ where on the whole, bilirubin^33^ (Scheme 1) is the most similar to molecule **1** in size and structure.

To study the interactions of **1** with DNA/RNA, several typical types of DNA and RNA were chosen (Supporting Information, Table S1). Naturally occurring calf thymus (ct)‐DNA represents a typical β‐helix structure with a balanced ratio of GC‐(48 %) and AT‐(52 %) base pairs. Synthetic alternating polynucleotides poly (dGdC)_2_ and poly (dAdT)_2_ represent two extreme situations (only AT‐ or GC‐), differing significantly in their secondary structure as well as in the availability of the minor groove for small‐molecule binding (the guanine amino group sterically hinders deep molecule penetration). For comparison between double stranded (ds) DNA and ds‐RNA, poly rA‐ poly rU was chosen as an α‐helical structure characterised by major groove available for binding of bulky small molecules.

Furthermore, to explore the DNA/RNA binding of the novel chromophore to a greater extent, we also studied the single‐stranded synthetic ss‐RNA polynucleotides poly G, poly A, poly U and poly C, each of them characterised by different properties. Thus, adenine ss‐RNA mimics 50 to 250 adenine nucleotides at the 3’ end of mRNA, poly G is related to guanine‐rich sequences in both DNA and RNA, whereas poly C and poly U are significantly more flexible than purine‐RNAs, and with less organised secondary structures.

Due to the possibility that **1** could interact with proteins, we examined the most naturally abundant protein, bovine serum albumin (**BSA**), taking into account its versatility of binding sites.

## Results and Discussion

Compound **1** was previously spectroscopically characterised in an aqueous medium.^3^ For studies of its interactions with DNA/RNA/protein we prepared a stock solution in water of 5 mm concentration, stable at low temperature in the dark for a long period, and diluted it with corresponding aqueous buffer prior to further experiments.

### Thermal denaturation experiments

It is well known that upon heating ds‐helices of polynucleotides, at a well‐defined temperature (*T*_m_ value) they dissociate into two single‐stranded polynucleotides. Non‐covalent binding of small molecules to ds‐polynucleotides usually increases the thermal stability of ds‐helices thus giving increased *T*_m_ values, whereby the increase (Δ*T*_m_) can (in corroboration with other methods) be related to the various binding modes.^34^

Compound **1** strongly stabilised all ds‐DNAs and ds‐RNA against thermal denaturation (Table 1) with no significant selectivity. The thermal stabilisation is in the range of many intercalators and groove binders, thus not giving a definite indication of the binding mode.^23^, ^34^, ^35^

**Table 1:** The Δ*T*_m_^[a]^ values (°C) of studied ds polynucleotides upon addition of ratio *r* ^[b]^ of **1** at pH 7.0 (sodium cacodylate buffer, *I*=0.05 mol dm^−3^).

*r* ^[b]^	ct‐DNA	poly(dA‐dT)_2_	poly A‐poly U
0.1	+7.3	+10.0	+9.5

[a] Error in Δ*T*_m_=±0.5 °C. [b] *r*=[compound][polynucleotide].

### Spectrophotometric titrations

Preliminary UV/Vis titration of **1** with ct‐DNA revealed a strong bathochromic (Δ$\tilde{\nu }$ =−956 cm^−1^) and pronounced hypochromic effect (Figure S1, Supporting Information); however, systematic deviation from the isosbestic point suggested more complexes formed, at least under UV/Vis titration conditions. Given that **1** is, in part of the titration, in excess over DNA (ratio *r*_[**1**][DNA]_>0.3), that suggested the possible aggregation of **1** along the polynucleotide, combined with a single molecule of **1** binding to DNA in the part of titration at which DNA is in excess (*r*_[**1**][DNA]_<0.3).

**Figure 1:**
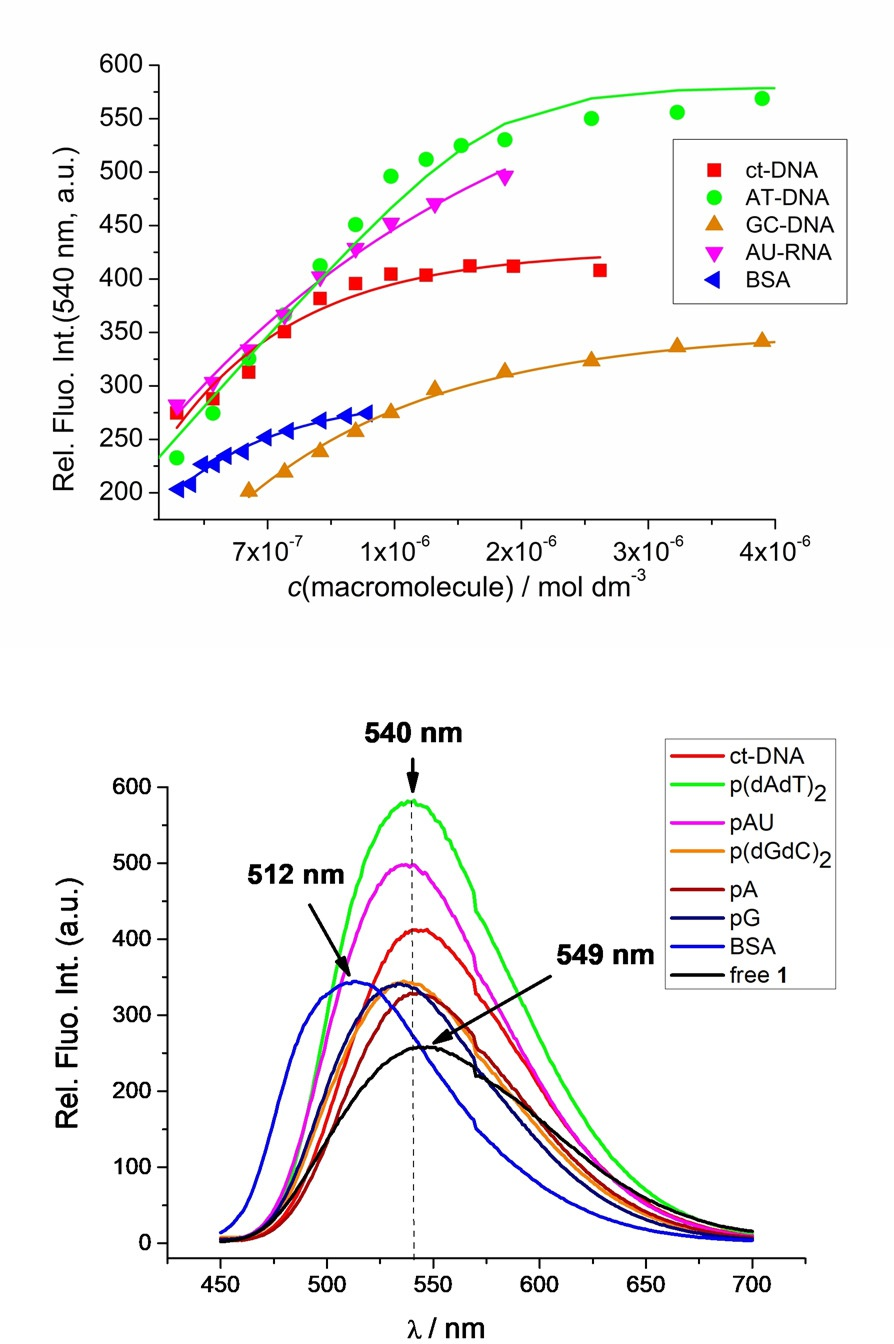
Top: Comparison of fluorimetric titrations of **1** (*c*=5×10^−7^ mol dm^−3^, *λ*_exc_=425 nm) at *λ*_em_=540 nm for all ds‐DNA, ds‐RNA and BSA (ss‐RNA not shown, see the Supporting Information). Bottom: Comparison of the fluorescence spectrum of **1** with spectra of **1**/biomacromolecule complexes at the end of the titrations. Note the significant hypsochromic shift of the **1**/BSA complex in comparison to the **1**/DNA, RNA complexes.

To ensure an excess of DNA over **1** throughout the titration (*r*_[**1**][DNA]_≪0.3), we took advantage of the intrinsic fluorescence of **1**, which allowed titrations at submicromolar concentrations. In general, upon addition of any DNA/RNA/protein, emission increased. However, the intensity of increase and, even more importantly, the shift of the emission maximum, strongly depended on the biomacromolecule (Figure 1). The magnitude of the emission increase was in the order AT‐DNA>AU‐RNA> mixed sequence ct‐DNA>GC‐DNA>ss‐RNAs and BSA (Figure 1). Intriguingly, addition of any DNA/RNA induced only a weak hypsochromic shift (Δ$\tilde{\nu }$ =304 cm^−1^) of the emission maximum, at variance with the strong hypsochromic shift (Δ$\tilde{\nu }$ =1360 cm^−1^) induced by the protein, BSA.

For all fluorimetric titrations performed (Figure 1 top, and Supporting Information), it was possible to calculate binding constants (Table 2); for DNA and RNA titrations, non‐linear fitting by means of the Scatchard equation (McGhee, von Hippel formalism)^36^, ^37^ was applied. The BSA titration data fit excellently to a 1:1 (**1**:BSA) stoichiometry model, pointing to only one dominant binding site of **1** at BSA.

**Table 2:** Binding constants (log*K*_s_) and spectroscopic properties of **1** with polynucleotides or BSA calculated by evaluation of fluorimetric titrations;^[a]^ *c*[**1**]=5×10^−7^ mol dm^−3^, at pH 7.0, sodium cacodylate buffer, *I*=0.05 mol dm^−3^.

Polynucleotide	Log*K*_s_^[a]^	*n* ^[a]^
ct‐DNA	7.0	0.25
poly dAdT–poly dAdT	7.9	0.25
poly dGdC–poly dGdC	7.6	0.25
poly A–poly U^[b]^	7^b^	0.25
poly G	6.9	0.5
poly A	6.6	0.5
poly C	6.9	0.5
poly U	6.4	0.5
BSA^[c]^	5.9^c^	1^c^

[a] Processing of titration data by means of the Scatchard equation^36^, ^37^ gave ratios of *n*[bound **1**][polynucleotide]=0.3–0.4. For easier comparison, all log*K*_s_ values were re‐calculated for fixed *n*=0.25 (ds‐polynucleotides) and *n*=0.5 (ss‐RNA). The accuracy of log*K*_s_ values is within an order of magnitude; thus, differences larger than ten‐fold can be considered as significant. Correlation coefficients were >0.99 for all calculated *K*_s_ values. [b] Fluorescence change was almost linear, thus allowing only estimation of the binding constant. [c] Fitted by non‐linear regression (software Origin 7.0) to 1:1 (**1**:BSA) stoichiometry model, *K*_s_ value error ±10 %.

The strong, submicromolar affinities of **1** toward ds‐DNA and ds‐RNA were within the same order of magnitude, in line with similar thermal stabilisations (Table 1), whereas its affinity toward ss‐RNA was approximately an order of magnitude lower. The affinity of **1** toward BSA was at least two orders of magnitude lower than toward ds‐DNA (Table 2). The excellent fit of the titration data (Figure S10, Supporting Information) strongly supported a single dominant binding site on BSA for **1**, although other binding sites of several orders of magnitude lower affinity cannot be excluded.

**Figure 2:**
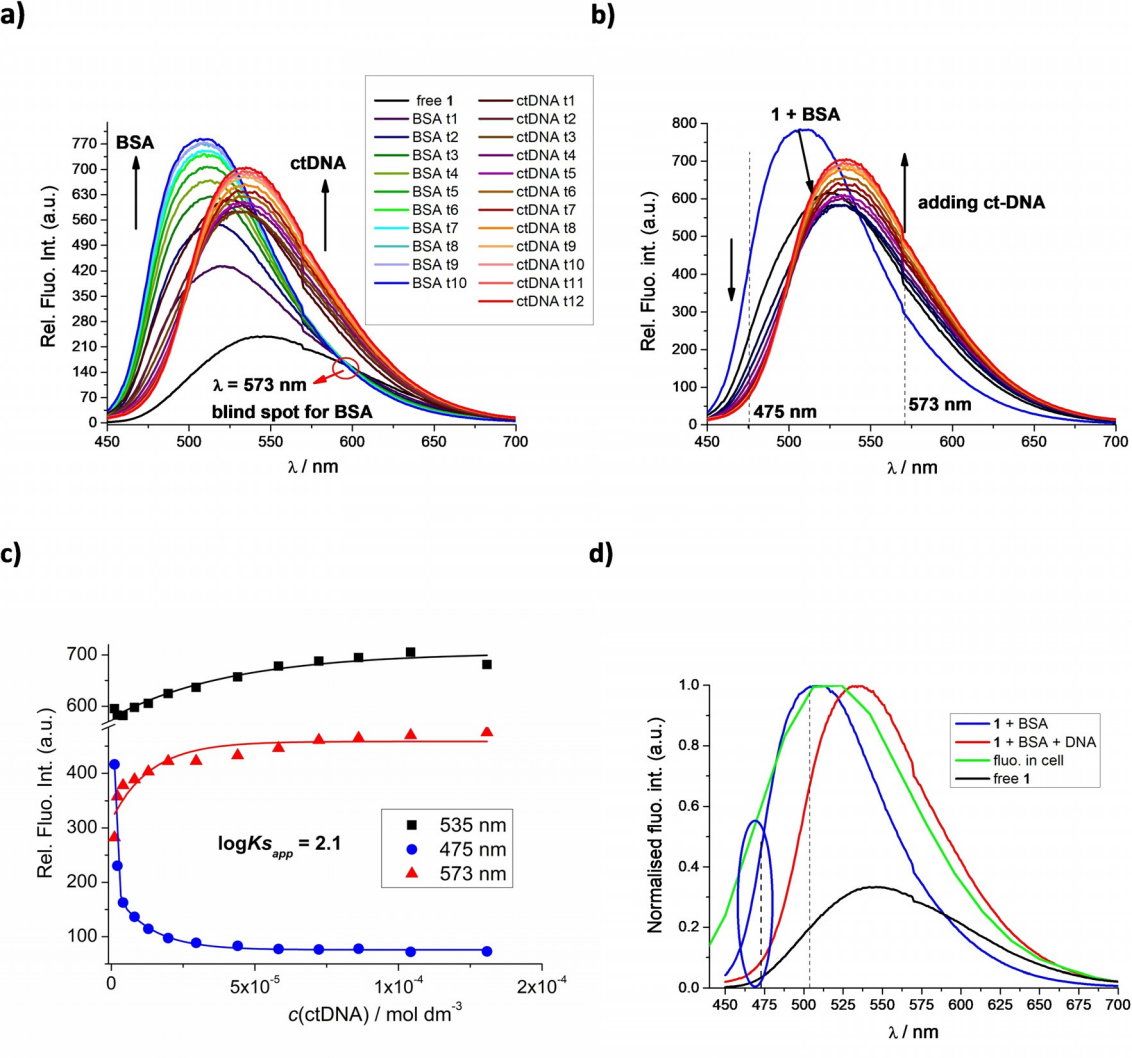
Competitive BSA/DNA experiment: a) the fluorimetric titration of **1** (*c*=5×10^−7^ mol dm^−3^ *λ*_exc_=425 nm) with BSA; the overlaid titration of **1** with ct‐DNA (ctDNA t1‐12) was done separately for comparison. Note the BSA isosbestic point (*λ*_em_=573 nm), at which fluorescence of **1** is not affected by any quantity of BSA (“blind spot”), but strongly changes upon addition of any DNA/RNA; b) the pre‐formed **1**/BSA complex was titrated with ct‐DNA; note the marked positions at λ_em_=475 nm and *λ*_em_=573 nm; c) the emission intensities at 475 nm (BSA characteristic), 573 nm (DNA characteristic) and 535 nm (**1**/DNA maximum) as a function of increasing *c*(ct‐DNA); d) overlaid emission (normalised to the intensity at *λ*_max_) of free **1**; **1**+BSA; **1**+BSA+DNA (last DNA addition from b) and fluorescence spectrum of **1** measured in the cell.^3^ Note in blue ellipsoid the emission area of **1** specific for protein (BSA).

The very intriguing difference between the emission spectra (Figure 1 bottom, Figure 2 a) of complexes **1**/BSA and **1**/DNA, RNA led us to perform a BSA/**1**/ds‐DNA competition experiment. Titration of **1** with BSA was performed (Figure 2 a; BSAt1‐t10) to ensure >90 % of the **1**/BSA complex formed, followed by titration with ct‐DNA (Figure 2 b). The typical **1**/BSA emission maximum (510 nm) clearly shifted toward the **1**/ds‐DNA maximum (540 nm), whereby non‐linear fitting of the titration data using the Scatchard equation^36^, ^37^ (see Table 2 for conditions) gave an apparent binding constant log*K*_s app_=2.1 (Figure 2 c), which agreed with the difference between log*K*_s_(**1**/ds‐DNA) and log*K*_s_(**1**/BSA).

Most importantly, by monitoring the changes of emission intensities at *λ*=475 (BSA dominant) and *λ*=573 nm (DNA specific), it was possible, by ratiometric analysis, to determine the percentage of both BSA and DNA in solution simultaneously. Thus, compound **1** could be considered to be a single‐molecule two‐target probe with well‐separated wavelengths at which each of these targets have a specific response (Δ$\tilde{\nu }$ =3600 cm^−1^). We applied these findings to the analysis of the previously reported^3^ fluorescence spectrum of **1** collected by confocal microscopy in cells (Figure 6 c in Ref. 3), whereby comparison with **1**/BSA and **1**/DNA spectra (Figure 2 d, particularly the blue ellipsoid area) clearly pointed to a protein‐like binding target as a dominant site in the cell.

### CD Experiments

Thus far, we had studied the non‐covalent interactions at 25 °C by monitoring the spectroscopic properties of compound **1** upon addition of the polynucleotides. In order to obtain insight into the changes of polynucleotide properties induced by the small‐molecule binding, we chose CD spectroscopy as a highly sensitive method for the examination of conformational changes in the secondary structure of polynucleotides.^38^ In addition, **1** as an achiral small molecule, can still generate an induced CD spectrum (ICD) upon binding to polynucleotides, which could give useful information about the modes of interaction.^39^, ^40^

**Figure 3:**
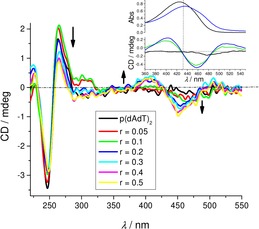
CD titration of poly (dAdT)_2_ (*c*=2×10^−5^ mol dm^−3^) with **1** at molar ratios *r*_[**1**][AT‐DNA]_=0–0.5. Inset: overlay of the UV/Vis and CD titration in the 360–550 nm range for *r*=0; 0.1; 0.2. Spectra were acquired at pH 7.0, using sodium cacodylate buffer, *I*=0.05 mol dm^−3^.

Addition of **1** to ds‐DNA/RNA resulted in a minor decrease of the intensity of the AT(U)‐containing polynucleotide CD spectra (230–300 nm range; Figures 3 and S14), whereas the GC‐DNA CD spectrum was only negligibly affected. AT‐Containing polynucleotides (Figures 3 and S14, Supporting Information, ct‐DNA) also revealed ICD bands at *λ*>300 nm, which could only be attributed to the uniformly oriented binding of **1** within a well‐defined DNA binding site.^40^ Closer inspection of the ICD bands and comparison with UV/Vis titration data (Inset Figure 3) revealed their bisignate nature with the zero point at the corresponding UV/Vis maximum (*λ*=432 nm), suggesting the DNA minor groove as the dominant binding site (see below). The lack of ICD bands for the **1**/GC‐DNA complex could be attributed to the sterically crowded minor groove with amino groups of guanine, not allowing deep penetration of **1** and thus diminishing the induced CD effect.^40^ The AU‐RNA broad and shallow minor groove is a poor binding site for small molecules, in contrast to the RNA major groove (width similar to DNA minor groove, Table S1), which could be an efficient binding site for **1**; however, the large depth of the major groove allowed a heterogeneous orientation of molecules of **1** with respect to the ds‐RNA chiral axis, thus inhibiting the appearance of measurable ICD bands.

**Figure 4:**
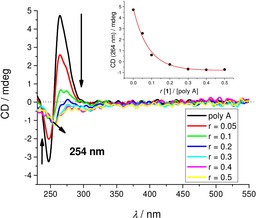
CD titration of poly A (*c*=2×10^−5^ mol dm^−3^) with **1** at molar ratios *r*_[**1**][poly A]_=0‐0.5 at pH 7.0, buffer sodium cacodylate, *I*=0.05 mol dm^−3^. Inset: dependence of CD band change at 264 nm on ratio *r*.

Particularly intriguing results were obtained for **1**/ss‐RNA complexes. Addition of **1** completely disordered the CD spectrum of poly A (Figure 4) and also those of poly C and poly U (Figure S14). In contrast to other ss‐RNAs, the CD spectrum of poly G was only marginally affected (Figure S14, Supporting Information), which could be attributed to the well‐known general stability of the poly G helical (and thus chiral) secondary structure in comparison to other ss‐polynucleotides.^41^, ^42^ For the poly A titration, the isoelliptic point at *λ*=254 nm strongly supported only one type of **1**/poly A complex, in contrast to poly U and poly C (systematic shifting of spectral cross points, Figure S14).

Complete loss of helical chirality of A, C, and U ss‐RNAs upon binding to **1**, accompanied with a rather high affinity and the fluorescence increase of **1** (Table 2, Figure 1 bottom) suggests wrapping of the ss‐polynucleotide chain around the cylindrical shape of **1**. Such a binding mode would maximize the efficiency of electrostatic interactions between the four positive charges of **1** and the negatively charged polynucleotide backbone, additionally supported by a strong hydrophobic effect of excluding the bis‐thiophene core from solution and also allowing eventual hydrogen bonding between RNA and thiophenes. Such a complex of achiral **1**, serving as a spindle for ss‐RNA, would not give any chiral response (in accordance with Figure 4).

### Molecular modelling of ligand 1 complexed in ds‐DNA and serum albumin

The above experimental results strongly support the binding of **1** within the ds‐DNA minor groove. Among the available single‐crystal X‐ray diffraction structures of typical minor groove binder/DNA complexes, the structure containing Hoechst 33258 was chosen as a starting point due to its similarity with **1** in terms of length and shape.

**Figure 5:**
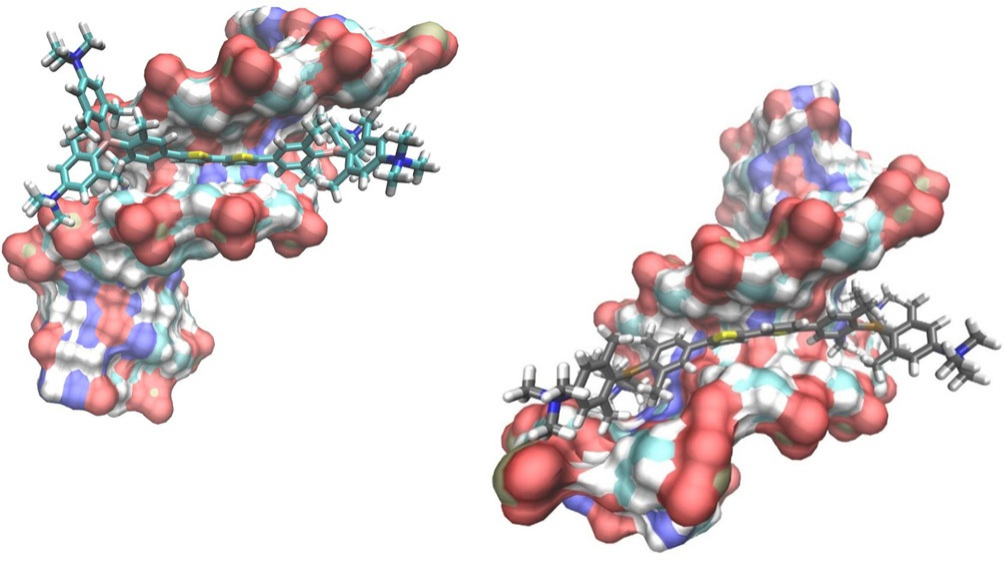
The result of 1 ns molecular dynamics simulation of **1**/DNA complexes; Left: *cis*; Right: *trans* configuration.

Thus, starting from the structure of Hoechst 33258 complexed in the minor groove of B‐DNA,^43^ docking was performed in PyMOL, wherein compound **1** was docked into the DNA minor groove using the position of Hoechst 33258 as a template. However, **1** can exist in two conformations (*cis* and *trans*) and in the crystal structure of a related compound^3^ the *trans* conformation was found. According to our DFT calculations the *trans* isomer is about 3.5 kcal mol^−1^ lower in energy than the *cis* isomer, and the energy barrier for the bond rotation between the two thiophenes is approximately 5.3 kcal mol^−1^ (see Figure S15, Supporting Information). Thus, it is feasible that **1** could adopt either conformation upon binding to DNA. Therefore, for each configuration of compound **1** (*cis* and *trans*) separate **1**/DNA complexes were considered, energy minimised, equilibrated and simulated for 1 ns,^44^, ^45^, ^46^, ^47^ as shown in Figure 5.

There are no significant energy differences between **1**_*cis*_/DNA and **1**_*trans*_/DNA complexes when compound **1** is accommodated within the DNA groove, and the energies of complex formation are similar to the experimentally determined values derived from the binding constants listed in Table 2.

In the resulting complexes (Figure 5), the DNA minor groove size and shape are not altered at the position of insertion of **1**, indicating that binding of **1** did not require a significant structural change of the ds‐DNA helix. In addition, all four positively charged phenyltrimethylammonium groups of **1** are positioned at ideal distances from the DNA backbone to form electrostatic interactions with negative phosphate groups. A closer inspection of the Van der Waals radii of **1** and its DNA binding site revealed an excellent fit, thus suggesting significant contributions of hydrophobic and Van der Waals interactions to the overall stability of the complex.

### Binding of 1 to bovine serum albumin (BSA)

**Figure 6:**
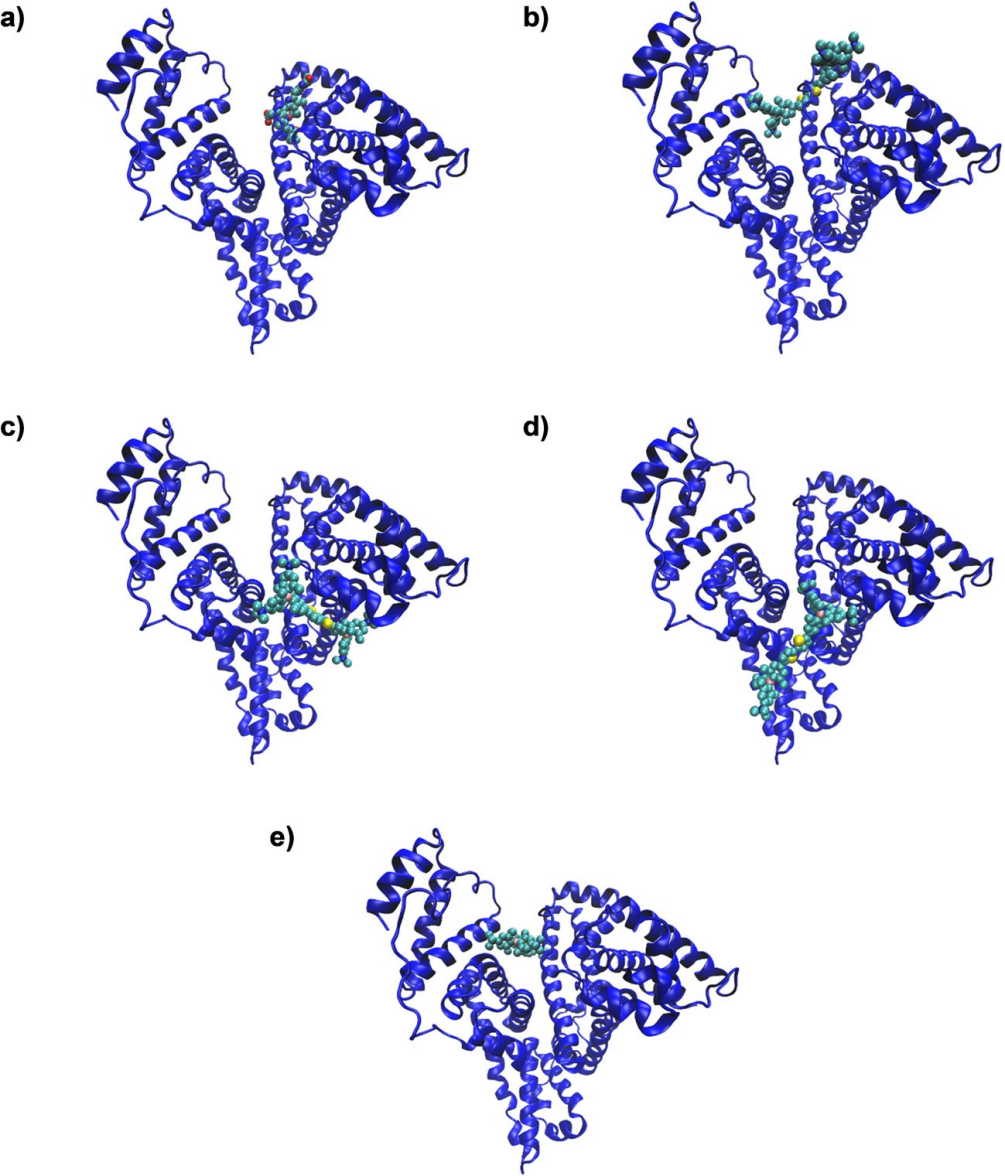
a) Starting X‐ray structure HSA/bilirubin;^33^ b) manually docked structure of **1** into the bilirubin binding site, followed by MM optimisation; c–e) set of the high scored structures the complex obtained by AUTODOCK4.^48^

The binding site(s) of **1** to BSA or HSA were not experimentally determined; however, an excellent fit of the titration data to 1:1 **1**/BSA stoichiometry (Figure S10, Supporting Information) supported a single dominant binding site for **1** (although other binding sites of significantly lower affinity cannot be excluded). According to the literature, most BSA/HSA ligands are of low molecular weight (M_w_<500), with the exception of bilirubin and hemin (M_w_>700), which bind to the IB:FA1 position of HSA, characterised by a deep hydrophobic cleft.^32^ Both, bilirubin and hemin are similar to **1** insofar as they contain repetitive five‐membered heterocycles, as well as by approximate mass and volume. Furthermore, among all small molecules which bind to HSA/BSA, hematin and bilirubin show by far the highest affinities (log*K*_s_≈8 m ^−1^),^32^ which agree well with the binding constant determined for **1** (Table 2). Therefore, we used the experimentally determined single‐crystal X‐ray diffraction structure of human serum albumin complexed with 4*Z*,15*E*‐bilirubin‐IX‐alpha as a starting point (Figure 6 a).^33^

Bilirubin was removed from the complex and **1** was docked in two different ways: a) manually docked structure of **1** using the position of bilirubin as a template, followed by 10 ns MM optimisation (Figure 6 b); or b) along the IB:FA1 position of HSA, using AUTODOCK4.^48^ Eleven distinct clusters were generated and the representative structures of the three selected, high scored structures are shown in Figure 6 c–e. The selected structures are characterised by their mean binding energies ranging from −6 to −9 kcal mol^−1^, corresponding nicely to values of the experimentally determined binding constant log *K*_s_≈6 m ^−1^ (Table 2). The analysis of these results (Figure 6) suggests that the proposed binding site is well‐suited for the accommodation of **1**.

## Conclusion

Our water‐soluble tetracationic quadrupolar bis‐triarylborane **1**, recently reported as a novel chromophore for one‐ and two‐photon excited fluorescence imaging in cells,^3^ showed strong non‐covalent interactions with ds‐DNA, ds‐RNA and also ss‐RNA, as well as one of the most abundant protein carriers, bovine serum albumin (BSA). The most intriguing advantage of such a broad set of targets is the selective fluorimetric response of **1** upon binding to DNA/RNA (at *λ*_em_=573 nm) with respect to BSA (at *λ*_em_=475 nm), whereby the separation of almost 100 nm between the characteristic emissions allows for the simultaneous ratiometric determination of both DNA/RNA and BSA in solution. Importantly, we demonstrated the applicability of this approach by analysing fluorescence spectra of **1** previously collected in vitro (in cell, Figure 2 d and Ref. 3), clearly defining protein and not DNA as a main target. This novel finding suggests a number of new possible biochemical and biomedical applications for **1** as a multipurpose simultaneous fluorescent sensor.

Furthermore, **1** binds to ds‐DNA/RNA as a groove binder but, in contrast with most groove binders,^49^, ^50^ it also interacts strongly with ss‐RNA, by acting as a spindle around which ss‐RNAs are wrapped due to a combination of electrostatic, H‐bonding and hydrophobic interactions. As most small molecule interactions with ss‐RNA are based on an intercalative binding mode, often combined with additional H‐bonding, whereby helicity of ss‐RNA is preserved^51^, ^52^ or even enhanced,^53^ the binding mode of **1** presented herein is unique. Such ss‐RNA condensation due to complexation (extended strand to globular transformation) could protect its backbone against RNAase degradation and also facilitate its cellular uptake and distribution, opening new possibilities for RNA‐based applications.

To the best of our knowledge, the multifaceted binding activity of a small molecule toward DNA/RNA and proteins presented here, and the selectivity of its fluorimetric and chirooptic response is unmatched in the literature. Thus, our quadrupolar bis‐triarylborane could be considered as a novel chromophore/fluorophore moiety for biochemical applications. The effect of systematic variations of the properties of the linker connecting the two triarylborane units, as well as modifying the number of positive charges at the termini, are the subject of ongoing investigations, the results of which will be presented in due course.

## Experimental Section

### Materials and methods

The synthesis and characterisation of **1** were reported previously.^3^ All measurements were performed in aqueous buffer solution (pH 7.0, *I*=0.05 m, sodium cacodylate/HCl buffer). The UV/Vis spectra were recorded on a Varian Cary 100 Bio spectrometer and fluorescence spectra were recorded on a Varian Cary Eclipse fluorimeter in quartz cuvettes (1 cm). Under the experimental conditions used, the absorbance of **1** was proportional to its concentration.

Polynucleotides were purchased as noted: poly dGdC—poly dGdC, poly dAdT—poly dAdT, poly A—poly U, poly A, poly G, poly C, poly U (Sigma), calf thymus (ct)‐DNA (Aldrich) and dissolved in sodium cacodylate buffer, *I*=0.05 m, pH 7.0. The ct‐DNA was additionally sonicated and filtered through a 0.45 mm filter to obtain mostly short (approx. 100 base pairs) rod‐like B‐helical DNA fragments.^54^ The polynucleotide concentration was determined spectroscopically^55^ as the concentration of phosphates (corresponds to *c*(nucleobase)).

Bovine Serum Albumin (BSA) (Sigma–Aldrich) was dissolved in sodium cacodylate buffer, *I*=0.05 m, pH 7.0 and its concentration determined spectroscopically using a NanoDrop spectrophotometer at 280 nm using its molar extinction coefficient of 43 824 m ^−1^ cm^−1^.

### Spectroscopic titrations

In fluorimetric experiments, an excitation wavelength of *λ*_exc_=425 nm was used to avoid absorption of excitation light by added polynucleotides or BSA. Fluorimetric titrations were performed by adding portions of polynucleotide or BSA solution into the solution of the studied compound being studied (*c*=5×10^−7^ m). After mixing polynucleotides or BSA with the compound, equilibrium was reached in less than 120 s. Fluorescence spectra were collected using an excess of DNA/RNA (*r*_[**1**][DNA]_<0.3) to assure one dominant binding mode. To obtain binding constants (*K*_s_), titration data were processed by means of non‐linear fitting to the Scatchard equation (McGhee, von Hippel formalism),^36^, ^37^ which gave values of the ratio of [bound compound][polynucleotide] in the range 0.1–0.3, but for easier comparison, all *K*_s_ values were re‐calculated for the fixed *n*=0.25 (for ds‐DNA/RNA) or 0.5 (for ss‐RNA). Calculated values for *K*_s_ have satisfactory correlation coefficients (>0.99). Titration data with BSA gave an excellent correlation (>0.999) to non‐linear regression fitting to a 1:1 (**1**:BSA) stoichiometry model, giving a value of *K*_s_.

CD Spectra were recorded on JASCO J‐815 spectropolarimeter at room temperature using 1 cm path quartz cuvettes with a scanning speed of 200 nm min^−1^ (an average of 3 accumulations). A buffer background was subtracted from each spectrum. CD experiments were performed by adding portions of compound stock solution into the solution of the polynucleotide (*c*=2×10^−5^ m).

Thermal melting experiments were performed on a Varian Cary 100 Bio spectrometer in quartz cuvettes (1 cm). The measurements were carried out in aqueous buffer solution at pH 7.0 (sodium cacodylate buffer, *I*=0.05 m). Thermal melting curves for ds‐DNA, ds‐RNA and their complexes with **1** were determined by monitoring the absorption change at 260 nm as a function of temperature.^34^ *T*_m_ values are the midpoints of the transition curves determined from the maximum of the first derivative and checked graphically by the tangent method. The Δ*T*_m_ values were calculated by subtracting *T*_m_ of the free nucleic acid from *T*_m_ of the complex. Every Δ*T*_m_ value here reported was the average of at least two measurements. The error in Δ*T*_m_ is ±0.5 °C.

### Molecular‐modelling methods

#### Docking

Compound **1** was docked into the single‐crystal X‐ray diffraction structure of human serum albumin (HSA) in two different ways: a) manually using the position of bilirubin in the structure of the HSA—bilirubin complex (PDB code 2VUE) as a template;^33^ or b) using the program AUTODOCK4.^48^

AUTODOCK4 procedure: The grid of 120×120×120 grid points with spacing of 0.375 Å centred at the protein's centre was used to generate atom‐specific affinity, electrostatic and desolvation maps. The docking procedure was performed using the Lamarckian genetic algorithm (LGA) in 20 copies, with a maximum of 2 500 000 energy evaluations. During docking, the protein was kept rigid whereas the ligand (compound **1**) was flexible.

### Molecular simulations

Compound **1** (its *cis* and *trans* form) was optimized using Gaussian, version g09‐D0.1 software, at the B3LYP/6‐31G(d) level of theory, and the parametrisation procedure was performed using the Antechamber^44^ module within the AMBER16 program suite wherein the Mullikan charges were used as the partial atomic charges.

Parameterisation, energy minimisation, and MD simulations of the complexes between compound **1** (both in *cis* and *trans* conformations) and macromolecules, DNA and serum albumin were performed using the AMBER16 suite of programs.^56^ The solutes were prepared using the AMBER16 utility program tLeap in association with the general AMBER force field gaff for ligand^57^ and ff14sb^58^ and ff99bsc0^59^ force fields for simulations of the DNA‐**1** and HSA‐**1** complexes, respectively. All Arg and Lys residues were positively charged and Glu and Asp negatively charged in HSA. Protonation states of histidines were determined manually, based on their ability to form hydrogen bonds with the neighbouring amino acids.

The systems were solvated in the truncated octahedron box filled with TIP3P water molecules^45^ whereas the sodium ions^46^ were added to achieve electroneutrality.

For each configuration of compound **1** (*cis* and *trans*) two complexes were examined. The complexes were minimised, equilibrated and simulated for 1 ns by the programs sander.MPI and pmemd.MPI. The programs used are part of the AMBER16 suite of programs. The simulations were performed using periodic boundary conditions (PBC). The particle mesh Ewald (PME) method was used for calculation of the long‐range electrostatic interactions, and in the direct space the pairwise interactions were calculated within the cut‐off distance of 10 Å. The solvated complexes were geometry optimised by using steepest descent and conjugate gradient methods, 1500 steps of each, and equilibrated for 130 ps. During the first stage of equilibration (30 ps), the temperature was linearly increased from 0 to 300 K and the volume was held constant. In the second stage, temperature and pressure were held fixed (300 K and 1 atm, respectively) and the solution density was optimised. The equilibrated complexes were subjected to productive molecular dynamics simulation using NPT conditions and a time step of 1 fs. The temperature was held constant using a Langevin thermostat^60^ with a collision frequency of 1 ps^−1^. Pressure was regulated by a Berendsen barostat.^61^

### DFT calculations

PES calculations were carried out with the program package Gaussian 09 (Revision D.01).^62^ Geometries were optimised without symmetry contraints using the B3LYP functional^63^, ^64^, ^65^, ^66^ in combination with a 6‐31G(d) basis set.^67^, ^68^ A relaxed potential‐energy surface scan was performed, during which the geometry was optimised at each point with a frozen dihedral angle (S‐C‐C‐S).

## Conflict of interest

The authors declare no conflict of interest.

## Supporting information

As a service to our authors and readers, this journal provides supporting information supplied by the authors. Such materials are peer reviewed and may be re‐organized for online delivery, but are not copy‐edited or typeset. Technical support issues arising from supporting information (other than missing files) should be addressed to the authors.

Supplementary
