# Supplementary material for: A Quadrupolar Bis‐Triarylborane Chromophore as a Fluorimetric and Chirooptic Probe for Simultaneous and Selective Sensing of DNA, RNA and Proteins
Source: Chemistry. 2020 Jan 22;26(10):2195–203. doi: 10.1002/chem.201903936 (PMC7065195; doi:10.1002/chem.201903936)
Supplement: Supplementary file 1 — Supplementary [file CHEM-26-2195-s001.pdf]

CHEMISTRY
A European Journal

Supporting Information

A Quadrupolar Bis-Triarylborane Chromophore as a Fluorimetric and Chirooptic Probe for Simultaneous and Selective Sensing of DNA, RNA and Proteins

Željka Ban,[a] Stefanie Griesbeck,[b] Sanja Tomić,[a] Jörn Nitsch,[b] Todd B. Marder,*[b] and Ivo Piantanida*[a]

chem_201903936_sm_miscellaneous_information.pdf

Supporting Information

A quadrupolar bis-triarylborane chromophore as a fluorimetric and chiroptic probe for simultaneous and selective sensing of DNA, RNA and proteins

Željka Ban,[a] Stefanie Griesbeck,[b] Sanja Tomić,[a] Jörn Nitsch,[b] Todd B. Marder,[b]* Ivo Piantanida[a]*

[a] Division of Organic Chemistry and Biochemistry, Ruđer Bošković Institute, Zagreb, Croatia, E-mail: pianta@irb.hr

[b] Institut für Anorganische Chemie, and Institute for Sustainable Chemistry & Catalysis with Boron, Julius-Maximilians-Universität Würzburg, Würzburg, Germany

Contents

General information 3
Spectrophotometric titrations 4
Thermal melting experiments 9
CD experiments 11
DFT-calculations 13
XYZ-Coordinates (DFT) 14
References 18

General information

Table S1. Structural properties of studied DNA and RNA.[1, 2]

Structure type	Groove width major	minor	Groove major	minor
**A_nU_n**	3.8	10.9	-	-
**[a]B-DNA**	11.7	5.7	8.5	7.5
**$(dGdC)_n$**	13.5	9.5	10.0	7.2
**$(dAdT)_n$**	11.2	6.3	-	-

[a] Calf Thymus (ct)-DNA

Spectrophotometric titrations

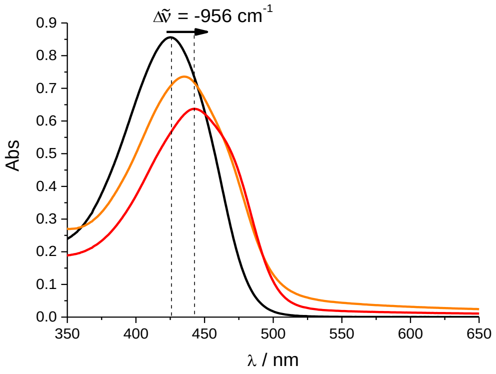

Figure S1. UV/Vis titration of **1**, $c = 2\times10^{-5}$ mol dm^{-3} with 5x and 20x ct-DNA, in buffered solution pH 7.0, (I = 0.05 mol dm^{-3}).

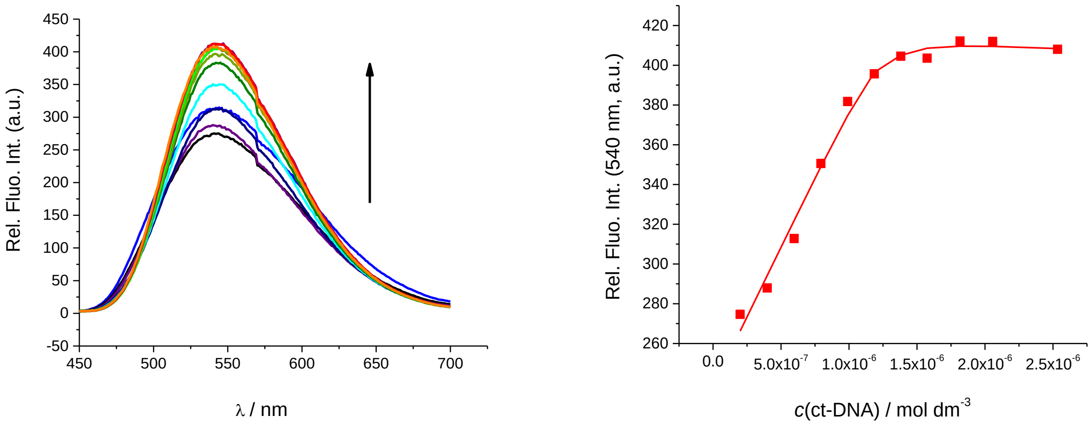

Figure S2. Left: Fluorimetric titration of **1** ($c = 5\times10^{-7}$ mol dm^{-3}; λ_{exc}= 425 nm) with **ctDNA** at pH 7, sodium cacodylate buffer, I = 0.05 mol dm^{-3}. Right: Dependence of fluorescence at λ_{max} = 540 nm on c(DNA), red line is non-linear least square fitting of Scatchard eq. (McGhee, von Hippel formalism)[3] to the experimental data.

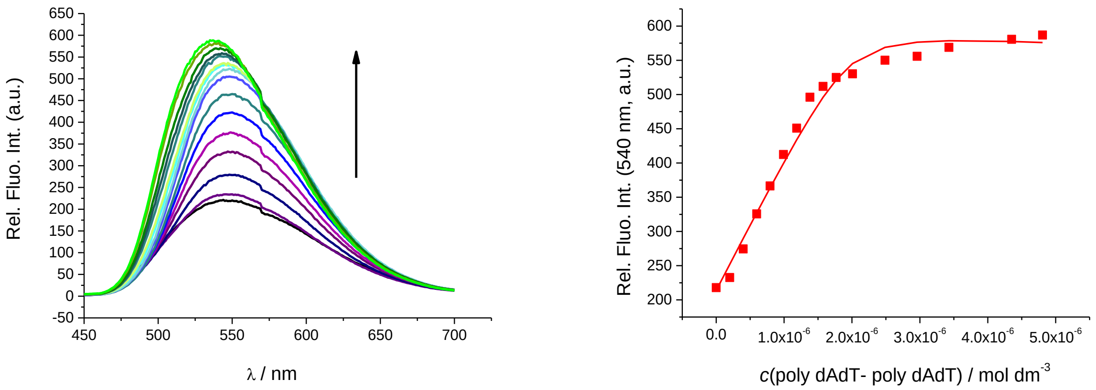

Figure S3. Left: Fluorimetric titration of **1** ($c = 5\times10^{-7}$ mol dm^{-3}; λ_{exc} = 425 nm) with **poly dAdT - poly dAdT** at pH 7, sodium cacodylate buffer, I = 0.05 mol dm^{-3}. Right: Dependence of fluorescence at λ_{max} = 540 nm on c(DNA), red line is the non-linear least square fitting of Scatchard eq. (McGhee, von Hippel formalism[3]) to the experimental data.

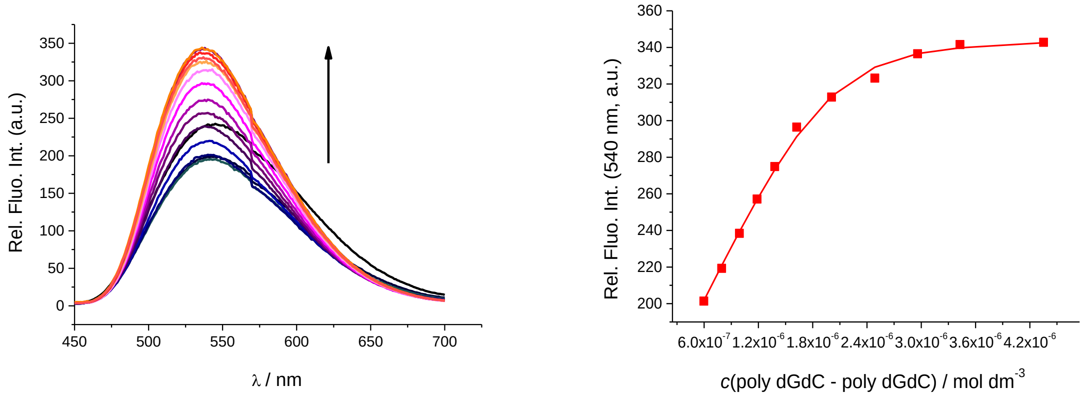

Figure S4. Left: Fluorimetric titration of **1** ($c = 5\times10^{-7}$ mol dm^{-3}; λ_{exc} = 425 nm) with **poly dGdC - poly dGdC** at pH 7, sodium cacodylate buffer, I = 0.05 mol dm^{-3}. Right: Dependence of fluorescence at λ_{max} = 540 nm on c(DNA), red line is the non-linear least square fitting of Scatchard eq. (McGhee, von Hippel formalism[3]) to the experimental data.

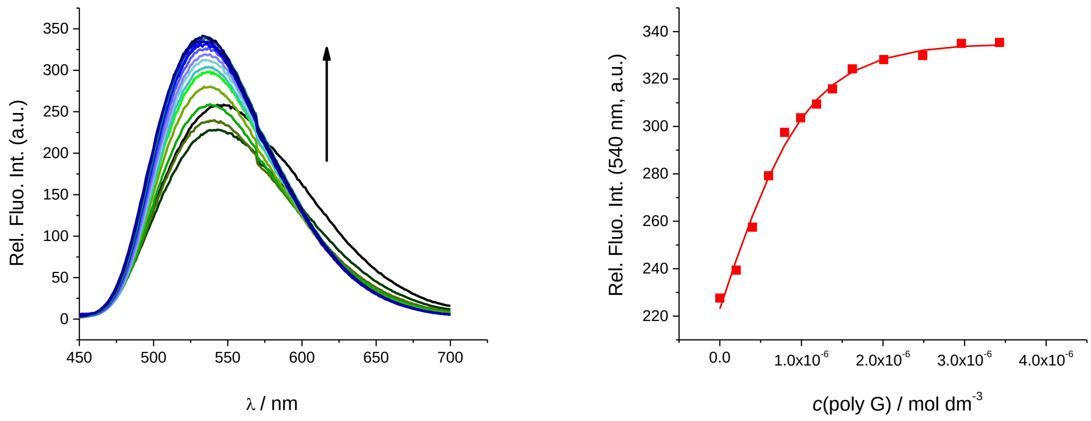

Figure S5. Left: Fluorimetric titration of **1** ($c = 5\times10^{-7}$ mol dm^{-3}; λ_{exc} = 425 nm) with **poly G** at pH 7, sodium cacodylate buffer, I = 0.05 mol dm^{-3}. Right: Dependence of fluorescence at λ_{max} = 540 nm on c(DNA), red line is the non-linear least square fitting of Scatchard eq. (McGhee, von Hippel formalism[3]) to the experimental data.

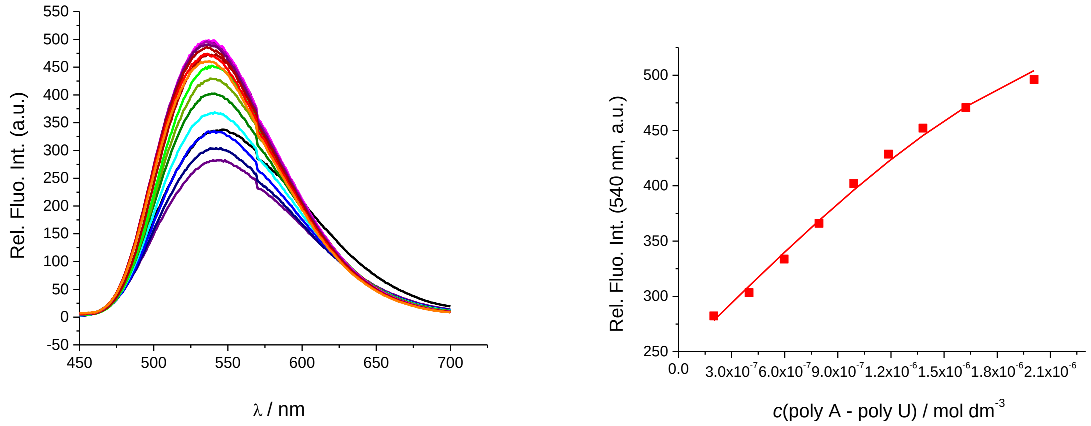

Figure S6. Left: Fluorimetric titration of **1** (c = 5×10^{-7} mol dm^{-3}; λ_{exc} = 425 nm) with **poly A – poly U** at pH 7, sodium cacodylate buffer, I = 0.05 mol dm^{-3}. Right: Dependence of fluorescence at λ_{max} = 540 nm on c(RNA), red line is the almost linear least square fitting of Scatchard eq. (McGhee, von Hippel formalism[3]) to the experimental data.

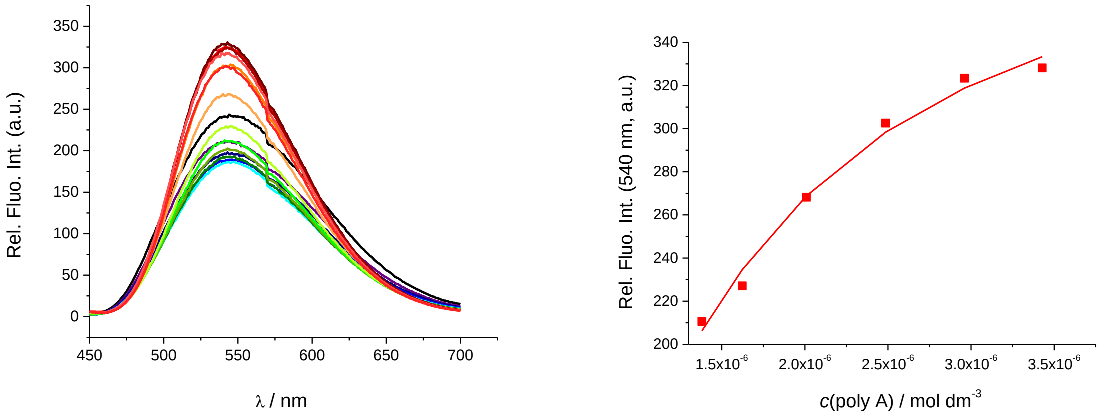

Figure S7. Left: Fluorimetric titration of **1** (c = 5×10^{-7} mol dm^{-3}; λ_{exc} = 425 nm) with **poly A** at pH 7, sodium cacodylate buffer, I = 0.05 mol dm^{-3}. Right: Dependence of fluorescence at λ_{max} = 540 nm on c(RNA), red line is the non-linear least square fitting of Scatchard eq. (McGhee, von Hippel formalism[3]) to the experimental data.

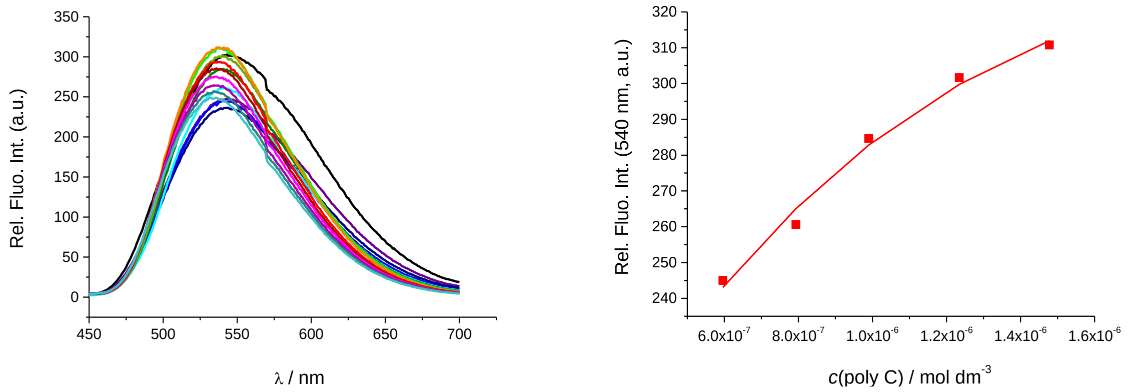

Figure S8. Left: Fluorimetric titration of **1** (c = 5×10^{-7} mol dm^{-3}; λ_{exc} = 425 nm) with **poly C** at pH 7, sodium cacodylate buffer, I = 0.05 mol dm^{-3}. Right: Dependence of fluorescence at λ_{max} = 540 nm on c(RNA), red line is the non-linear least square fitting of Scatchard eq. (McGhee, von Hippel formalism[3]) to the experimental data.

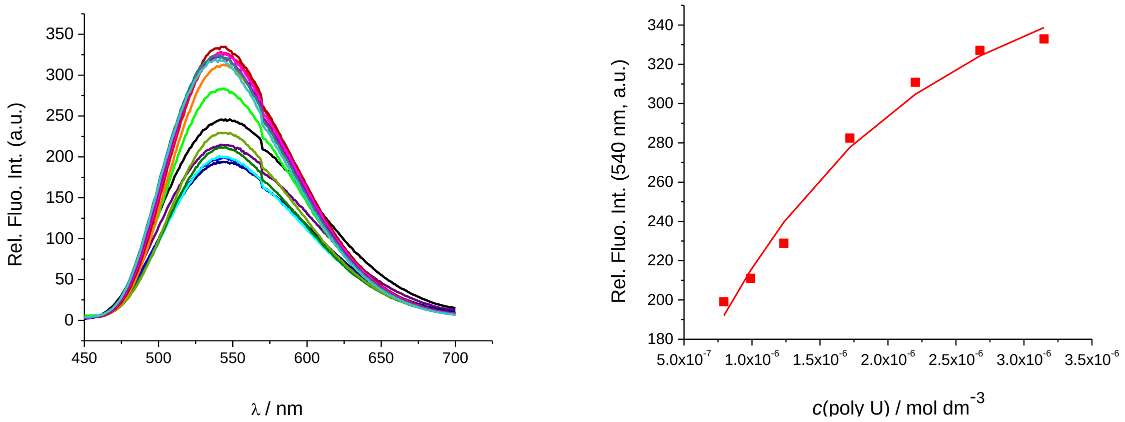

Figure S9. Left: Fluorimetric titration of **1** (c = 5×10^{-7} mol dm^{-3}; λ_{exc} = 425 nm) with **poly U** at pH 7, sodium cacodylate buffer, I = 0.05 mol dm^{-3}. Right: Dependence of fluorescence at λ_{max} = 540 nm on c(RNA), red line is the non-linear least square fitting of Scatchard eq. (McGhee, von Hippel formalism[3]) to the experimental data.

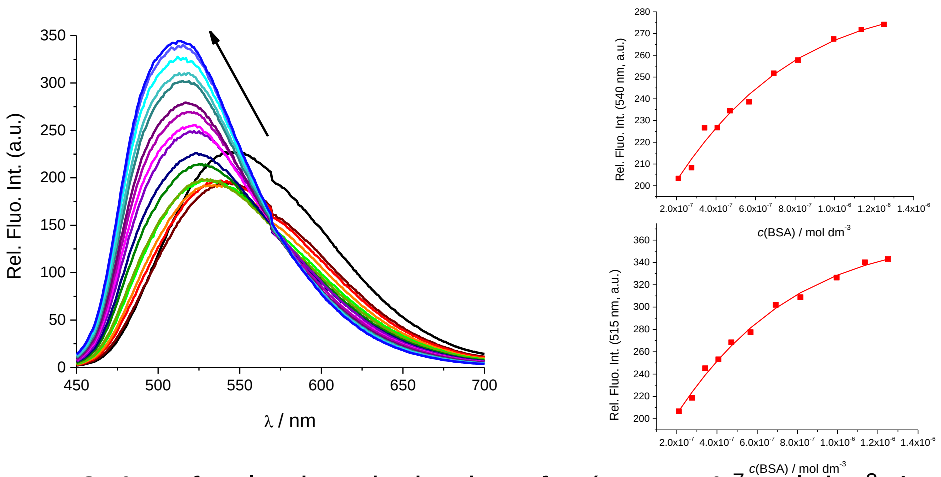

Figure S10. Left: Fluorimetric titration of **1** (c = 5×10^{-7} mol dm^{-3}; λ_{exc} = 425 nm) with **BSA** (bovine serum albumin) at pH 7, sodium cacodylate buffer, I = 0.05 mol dm^{-3}. Right: Dependence of fluorescence at λ_{max} on c(BSA) (at λ_{max}=545 nm TOP; at λ_{max}=515 nm DOWN) red line is the non-linear least square fitting to the experimental data for 1:1=**1**/BSA stoichiometry.

Thermal melting experiments

It is well known that, upon heating, ds-helices of polynucleotides at a well-defined temperature (*Tm* value) dissociate into two single stranded polynucleotides. Non-covalent binding of small molecules to ds-polynucleotides usually has an effect on the thermal stability of helices thus giving different *Tm* values. The difference between the *Tm* value of the free polynucleotide and the complex with a small molecule (Δ*Tm* value) is an important factor in the characterisation of small molecule / ds-polynucleotide interactions.[4]

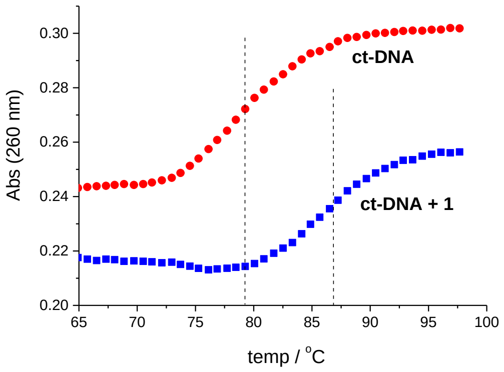

Figure S11. Thermal denaturation curves of ct-DNA (c(ct-DNA) = 2.5 × 10^{-5} M, $r_{[\mathbf{1}]/[\text{ct-DNA}]}$ = 0.1) at pH 7.0 (sodium cacodylate buffer, *I* = 0.05 M) upon addition of **1**. Error in ΔTm values: ±0.5 °C.

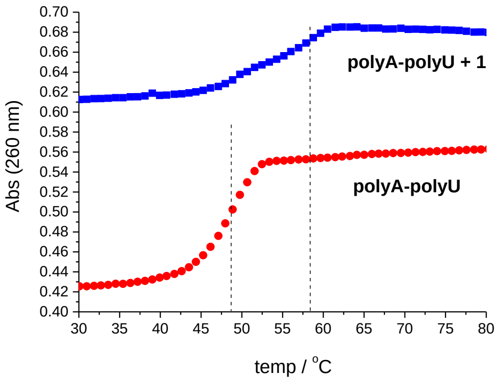

Figure S12. Thermal denaturation curves of **poly A – poly U** (*c*(**poly A – poly U**) = 2.5 × 10^{-5} M, $r_{[\mathbf{1}]/[\text{poly A – poly U}]}$ = 0.1) at pH 7.0 (sodium cacodylate buffer, *I* = 0.05 M) upon addition of **1**. Error in ΔTm values: ±0.5 °C.

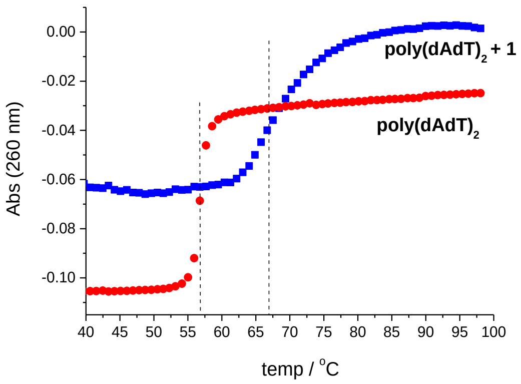

Figure S13. Thermal denaturation curves of **poly (dAdT)$_2$** (c(**poly (dAdT)$_2$**) = 2.5 × 10^{-5} M, $r_{[\mathbf{1}]/[\textbf{poly (dAdT)2}]}$ = 0.1) at pH 7.0 (sodium cacodylate buffer, I = 0.05 M) upon addition of **1**. Error in ΔTm values: ±0.5 °C.

CD experiments

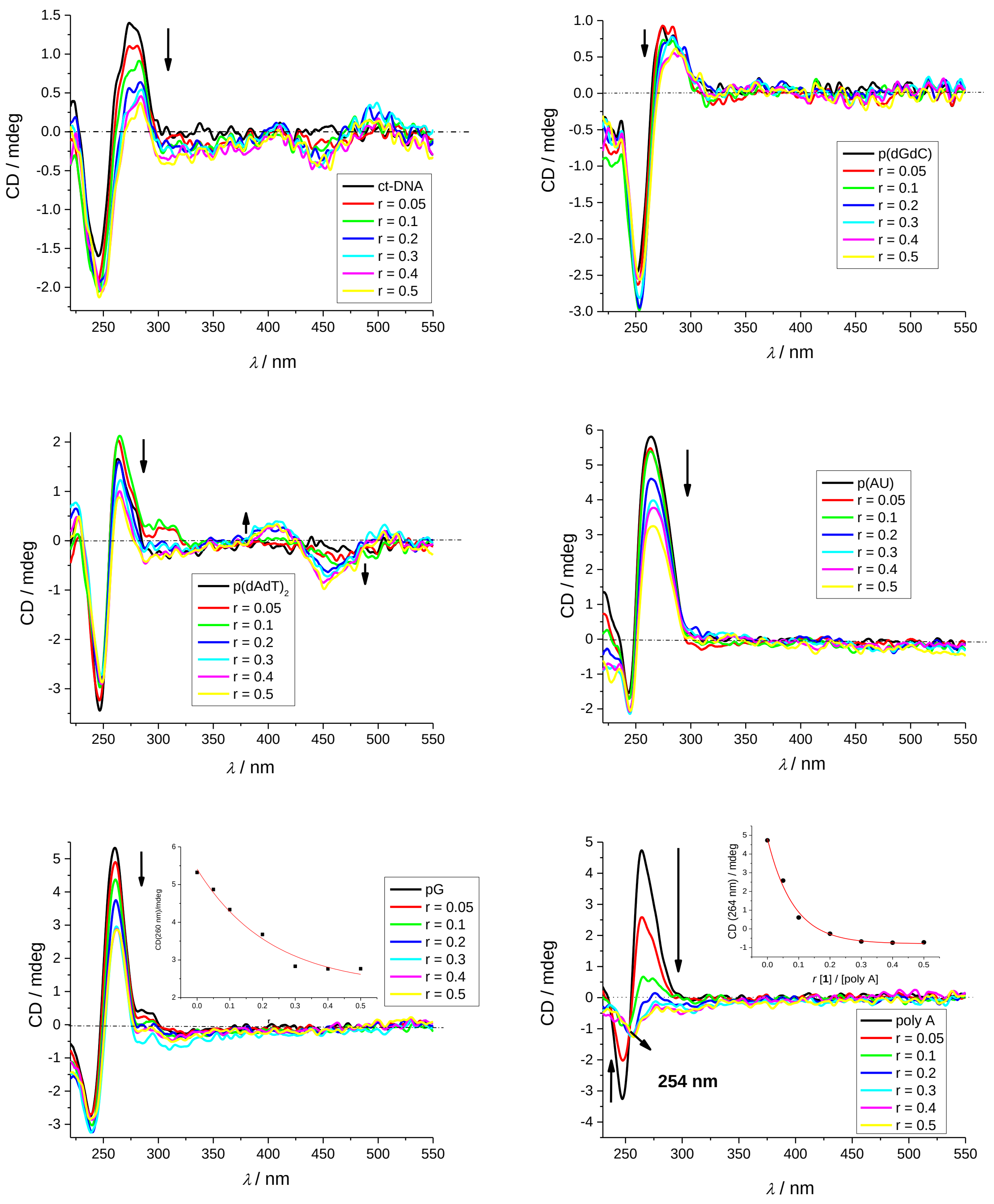

Figure S14. CD titration of **ctDNA**, **poly (dGdC)₂**, **poly (dAdT)₂**, **poly A- poly U**, **pG**, **poly A**, **poly U** and **poly C** (all DNA/RNA $c = 2\times10^{-5}$ mol dm^{-3}) with **1** at molar ratios ***r*** = [compound] / [polynucleotide] (pH 7.0, buffer sodium cacodylate, I = 0.05 mol dm^{-3}).

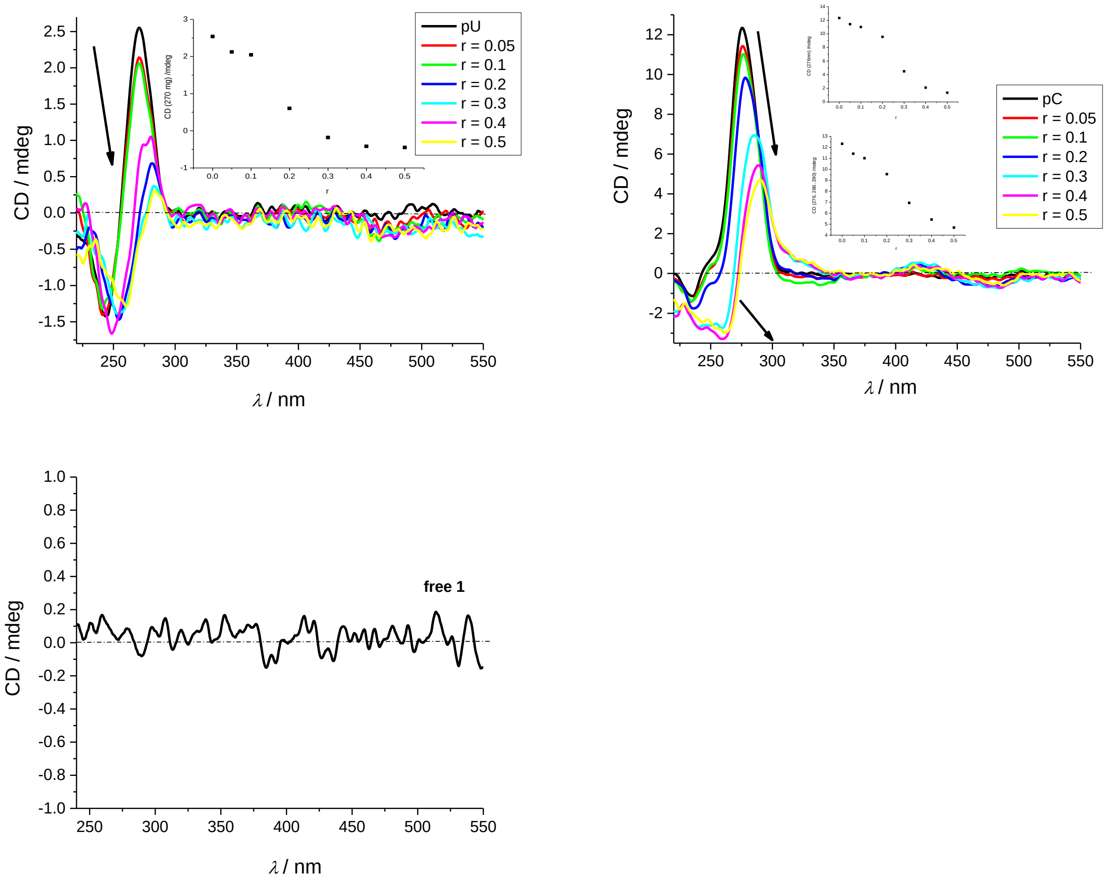

Figure S14 continued. CD titration of **ctDNA**, **poly (dGdC)$_2$**, **poly** (**dAdT)$_2$**, **poly A-poly U**, **pG**, **poly A**, **poly U** and **poly C** (all DNA/RNA *c* = 2×10^{-5} mol dm^{-3}) with **1** at molar ratios ***r*** = [compound] / [polynucleotide] (pH 7.0, buffer sodium cacodylate, *I* = 0.05 mol dm^{-3}).

DFT-calculations

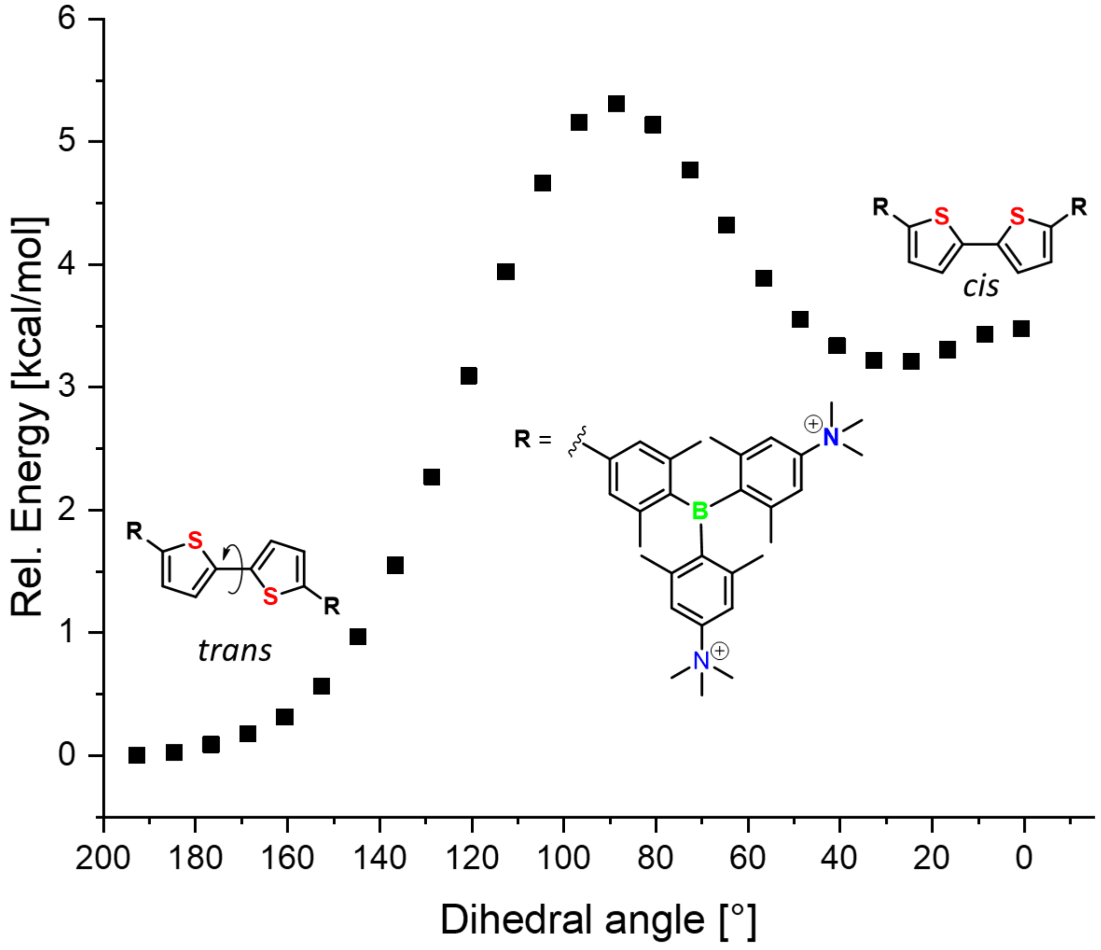

Figure S15. Potential energy surface (PES) scan of compound **1** interconverting the *trans* into the *cis* isomer by variation of the S-C-C-S dihedral angle.

XYZ-Coordinates (DFT)

***trans*-1**	***trans***		**[-3708.2517]**	***cis*-1**			**[-3708.2462]**
C	0.26384137	0.67275904	0.20084244	C	-0.02696019	0.72336701	-2.92620067
C	1.57847112	1.08850549	0.31600563	C	-0.13804895	1.53278288	-4.04500902
C	1.73441864	2.49138933	0.28220716	C	-0.17365631	2.90924539	-3.74813458
C	0.54725431	3.18693916	0.14456931	C	-0.09326033	3.19905777	-2.39844741
S	-0.79605354	2.06035743	0.0355642	S	0.02909205	1.707247	-1.47601964
H	2.40651441	0.39994515	0.44526275	H	-0.19657601	1.14252676	-5.05407906
H	2.69661166	2.97811269	0.39347995	H	-0.22882596	3.67342354	-4.51478115
C	-0.26384026	-0.67275892	0.20084238	C	0.02766307	-0.72332653	-2.92621953
C	-1.57847001	-1.08850537	0.31600555	C	0.13911856	-1.53269604	-4.04502805
S	0.79605464	-2.06035729	0.035564	S	-0.02887987	-1.70726597	-1.47610475
C	-1.73441754	-2.4913892	0.28220695	C	0.17462509	-2.90916733	-3.74820197
H	-2.4065133	-0.39994504	0.44526274	H	0.1979845	-1.14239608	-5.05406124
C	-0.54725323	-3.18693903	0.14456902	C	0.09377344	-3.1990461	-2.39855189
H	-2.69661057	-2.97811256	0.3934797	H	0.23005655	-3.67330096	-4.51487336
C	0.3479489	4.63249722	0.09391893	C	-0.08532364	4.51981201	-1.77727687
C	-0.90193342	5.22661264	0.33522303	C	0.42961202	4.75562364	-0.49169375
C	1.41966696	5.49458877	-0.19852412	C	-0.58968335	5.63009927	-2.47790141
H	-1.75468732	4.59323751	0.56485665	H	0.82252469	3.92128942	0.08371438
C	-1.08749706	6.60793664	0.31857495	C	0.4895224	6.03401804	0.06203346
C	1.26961451	6.87775465	-0.25735847	C	-0.5807781	6.91675099	-1.94706319
H	2.39705662	5.07045007	-0.40662256	H	-1.01987612	5.48006804	-3.46297396
C	0.00116951	7.48454876	0.01354664	C	-0.01702478	7.16443425	-0.6542251
C	-2.4951901	7.10075724	0.60196237	C	1.08468808	6.13941739	1.4545692
C	2.51991049	7.67584533	-0.58138292	C	-1.15535864	8.00931039	-2.83122929
B	-0.1834875	9.0384569	-0.01691959	B	0.06351324	8.61335955	-0.06921691
H	-2.57884456	7.54855079	1.59822704	H	2.08832697	6.57832282	1.43803842
H	-2.83447278	7.85119773	-0.11904537	H	0.47621383	6.75014027	2.12931991
H	-3.20469923	6.269723	0.55886194	H	1.1712935	5.14784897	1.90772178
H	2.48951079	8.09176076	-1.59421765	H	-2.1197752	8.37235954	-2.460232
H	2.67840651	8.51257172	0.10649242	H	-0.49456269	8.8778805	-2.91611081
H	3.405137	7.03688994	-0.51903785	H	-1.31876159	7.63045076	-3.84384112
C	-1.03059945	9.78967952	1.12001278	C	1.39516867	9.13823803	0.65588628
C	0.47397729	9.92570876	-1.18103183	C	-1.17384449	9.62717447	-0.20064165
C	-2.09616301	10.6709941	0.81011279	C	1.3736668	9.71761743	1.94888221
C	-0.70685532	9.59435839	2.49152781	C	2.64638945	9.06022762	-0.01707926
C	0.21699501	9.61106833	-2.54444679	C	-2.462182	9.23904693	0.26111096
C	1.30620011	11.0386917	-0.90368706	C	-1.03497814	10.9192542	-0.76601049
C	-2.81497746	11.3181331	1.82860614	C	2.55264683	10.1949565	2.54452729
C	-2.53657797	10.9428706	-0.61395246	C	0.10153077	9.83208947	2.76379727
C	-1.42021969	10.2696287	3.4892182	C	3.80500647	9.56525597	0.58550273
C	0.39953507	8.66573556	2.95161431	C	2.80561734	8.44309164	-1.39258559

C	0.76925098	10.3923545	-3.56658126	C	-3.54647689	10.1183438	0.15550045
C	-0.64476413	8.43827433	-2.96845161	C	-2.73802973	7.88091416	0.87517732
C	1.87075526	11.7928192	-1.94556153	C	-2.14210979	11.7754927	-0.88521712
C	1.65616715	11.459938	0.50912296	C	0.28610151	11.4387048	-1.29619819
C	-2.47251809	11.119787	3.15965664	C	3.75873301	10.1246318	1.85960501
H	-3.63034543	11.9676045	1.53793854	H	2.48255128	10.6157473	3.53911243
H	-2.72742077	10.0174994	-1.16738679	H	-0.42171605	8.87327083	2.84116898
H	-3.46039573	11.5276581	-0.63701226	H	0.31346419	10.1664611	3.78325887
H	-1.77604645	11.501383	-1.16861218	H	-0.59676261	10.5474297	2.31802798
H	-1.13325304	10.1020056	4.52221299	H	4.73612376	9.49954221	0.03216101
H	1.32317081	8.79856328	2.38130385	H	2.04485752	8.78775023	-2.09851816
H	0.63779267	8.8345421	4.00582813	H	3.78317069	8.68660308	-1.81880237
H	0.10823189	7.61656292	2.83811228	H	2.72178478	7.3524196	-1.34903515
C	1.59836105	11.470645	-3.26846854	C	-3.38899	11.374634	-0.42441693
H	0.53964745	10.1245814	-4.59280691	H	-4.51022856	9.7856375	0.52707231
H	-0.12612926	7.48763291	-2.8079323	H	-2.73084476	7.09368812	0.11448462
H	-0.90032197	8.50489875	-4.02999105	H	-3.71628446	7.86471636	1.36451522
H	-1.58251454	8.38328435	-2.40825852	H	-1.99174902	7.60157006	1.62445533
H	2.51412007	12.6211719	-1.67904714	H	-1.98546033	12.7433482	-1.34321779
H	0.78402837	11.8621137	1.0342467	H	0.99723979	11.6241445	-0.48496768
H	2.03595869	10.6233494	1.10455383	H	0.75841638	10.73241	-1.98641092
H	2.42969634	12.2329777	0.51044974	H	0.15094478	12.3779925	-1.83971625
N	-3.22180675	11.8087839	4.28181902	N	5.04616957	10.6449433	2.4657327
N	2.18779849	12.2663709	-4.41501651	N	-4.60273309	12.2737141	-0.54126196
C	-3.84079762	10.7689346	5.19753251	C	6.05116444	9.51402929	2.58170686
C	-2.26683669	12.6790357	5.07782545	C	5.61943047	11.7423368	1.58797743
C	-4.33831617	12.6971723	3.78512205	C	4.85358953	11.2239332	3.84770253
C	3.03787825	11.3578268	-5.28406982	C	-5.66189223	11.5938085	-1.38960683
C	1.07222234	12.8663084	-5.25064004	C	-5.16621211	12.5589531	0.83835915
C	3.06846404	13.4029135	-3.95086204	C	-4.29013536	13.6034564	-1.18720839
H	-3.05451539	10.1505826	5.62604751	H	6.26304309	9.11567005	1.59142907
H	-4.38393958	11.2844908	5.99128904	H	6.96577699	9.90670902	3.02909088
H	-4.5199341	10.1533216	4.60732159	H	5.6203642	8.73623303	3.21257968
H	-1.83253013	13.4173342	4.40342119	H	4.88332155	12.5432176	1.51564489
H	-2.82729864	13.1720903	5.87381243	H	6.5391363	12.1093575	2.04651963
H	-1.48220151	12.0579998	5.50553073	H	5.8317294	11.3410226	0.59900641
H	-3.92293645	13.4779878	3.14900048	H	4.15989456	12.0622909	3.79468488
H	-5.06198538	12.0954646	3.23633272	H	4.47445314	10.4499749	4.51414962
H	-4.81795082	13.1474176	4.65442948	H	5.82423164	11.571592	4.20192639
H	2.4173327	10.5611467	-5.68962964	H	-5.95821329	10.6593177	-0.91719304
H	3.46123653	11.9509361	-6.0963657	H	-6.52224961	12.2601625	-1.47074762
H	3.83053719	10.9356504	-4.66598789	H	-5.2376799	11.3962133	-2.3742679
H	0.4735174	13.513661	-4.60953145	H	-4.39144019	13.0450848	1.43162153
H	1.51796159	13.4412062	-6.06402763	H	-6.03231054	13.2140727	0.73126534

H	0.45373229	12.0677389	-5.65559704	H	-5.46392057	11.6237181	1.30838726
H	2.47927688	14.0941841	-3.34929032	H	-3.553518	14.1336413	-0.58454302
H	3.90186072	13.0022981	-3.37479964	H	-3.91603609	13.4359451	-2.19658422
H	3.44576767	13.9153505	-4.83602248	H	-5.21480045	14.1794244	-1.22871167
C	-0.34794783	-4.63249709	0.09391851	C	0.0856343	-4.51981422	-1.77743183
C	0.90193449	-5.22661253	0.33522252	C	-0.42942505	-4.7556109	-0.49189
C	-1.4196659	-5.49458856	-0.19852459	C	0.5899264	-5.63014689	-2.47803594
H	1.7546884	-4.59323742	0.56485615	H	-0.82230196	-3.92125278	0.08350797
C	1.08749813	-6.60793648	0.31857433	C	-0.48949302	-6.03400163	0.06181612
C	-1.26961346	-6.87775449	-0.25735903	C	0.58086929	-6.91680258	-1.94721515
H	-2.39705557	-5.07044988	-0.40662298	H	1.02017733	-5.48016218	-3.46308952
C	-0.00116847	-7.4845486	0.01354601	C	0.01701538	-7.16446452	-0.65441365
C	2.49519118	-7.10075719	0.60196164	C	-1.0847858	-6.13934913	1.45430218
C	-2.51990946	-7.67584512	-0.58138351	C	1.15541618	-8.0093839	-2.83137802
B	0.1834885	-9.03845679	-0.01692031	B	-0.06359709	-8.61335955	-0.0693884
H	2.57884573	-7.54855063	1.59822632	H	-2.08849321	-6.57809723	1.43767011
H	2.83447378	-7.85119768	-0.11904609	H	-0.47647052	-6.75018462	2.12908915
H	3.20470032	-6.2697229	0.5588611	H	-1.1712798	-5.1477775	1.90746951
H	-2.48950974	-8.0917606	-1.59421823	H	2.11995084	-8.37226598	-2.46051088
H	-2.67840553	-8.51257145	0.10649184	H	0.49472525	-8.87804512	-2.91605818
H	-3.40513596	-7.03688973	-0.51903848	H	1.31859989	-7.63059967	-3.84405372
C	1.03059996	-9.78967952	1.12001234	C	-1.39523474	-9.13816056	0.65582091
C	-0.47397684	-9.9257086	-1.18103223	C	1.17369281	-9.62727078	-0.20083855
C	2.09616353	-10.6709942	0.81011274	C	-1.3737198	-9.71745746	1.94884711
C	0.7068553	-9.59435845	2.49152726	C	-2.64646438	-9.0601782	-0.01713393
C	-0.21699488	-9.61106844	-2.54444731	C	2.46205007	-9.23918484	0.26089842
C	-1.30619987	-11.0386913	-0.90368706	C	1.03478778	-10.9193541	-0.76620303
C	2.81497753	-11.3181334	1.82860634	C	-2.55269571	-10.194748	2.54454026
C	2.53657904	-10.9428707	-0.61395235	C	-0.10155957	-9.83194818	2.76372102
C	1.42021921	-10.2696289	3.4892179	C	-3.8050793	-9.56515157	0.58549564
C	-0.39953519	-8.66573556	2.95161337	C	-2.8056728	-8.44316816	-1.392697
C	-0.76925134	-10.3923546	-3.56658151	C	3.5463155	-10.118524	0.15530127
C	0.64476442	-8.43827475	-2.96845257	C	2.73797398	-7.88105021	0.87493146
C	-1.8707555	-11.7928188	-1.94556125	C	2.14188982	-11.7756289	-0.88539885
C	-1.65616663	-11.4599373	0.50912313	C	-0.28628736	-11.4387024	-1.2964925
C	2.47251766	-11.1197873	3.15965671	C	-3.75879099	-10.1244499	1.8596336
H	3.63034553	-11.9676047	1.53793902	H	-2.48258531	-10.6154861	3.53914717
H	2.72742213	-10.0174995	-1.16738659	H	0.42192179	-8.87323347	2.84073929
H	3.46039676	-11.5276582	-0.6370118	H	-0.31350496	-10.1659354	3.78330675
H	1.77604768	-11.501383	-1.16861239	H	0.59653238	-10.5476085	2.31814195
H	1.13325218	-10.1020058	4.52221258	H	-4.73620382	-9.49946638	0.03216221
H	-1.3231707	-8.79856307	2.38130249	H	-2.04494776	-8.78796619	-2.09859852
H	-0.63779328	-8.83454215	4.00582706	H	-3.78325163	-8.68664806	-1.81887301
H	-0.10823184	-7.61656292	2.83811158	H	-2.72175454	-7.35249755	-1.34926162

C	-1.59836159	-11.4706449	-3.26846839	C	3.38878596	-11.3748108	-0.42460427
H	-0.53964805	-10.1245817	-4.59280726	H	4.5100779	-9.78584838	0.52687327
H	0.12612981	-7.48763317	-2.8079333	H	2.73083133	-7.09384418	0.11421785
H	0.90032199	-8.50489939	-4.02999205	H	3.7162327	-7.86489459	1.36426283
H	1.58251498	-8.38328488	-2.40825971	H	1.99171622	-7.60163425	1.62420489
H	-2.51412045	-12.6211714	-1.67904654	H	1.98520533	-12.7434872	-1.34338011
H	-0.78402779	-11.862113	1.03424672	H	-0.9979334	-11.6229535	-0.48545399
H	-2.03595782	-10.6233484	1.10455396	H	-0.75787955	-10.7328377	-1.98766179
H	-2.42969598	-12.2329768	0.51045026	H	-0.15133695	-12.3785895	-1.8390209
N	3.22180581	-11.8087843	4.28181935	N	-5.04622211	-10.6447146	2.46581027
N	-2.18779956	-12.2663708	-4.41501606	N	4.60249129	-12.2739431	-0.54142931
C	3.8407964	-10.7689351	5.19753315	C	-6.0512019	-9.51378216	2.58173883
C	2.26683535	-12.679036	5.07782536	C	-5.6195081	-11.7421476	1.58812204
C	4.33831535	-12.6971727	3.78512279	C	-4.85362185	-11.2236332	3.84780718
C	-3.03787934	-11.3578266	-5.28406926	C	5.66168099	-11.5940905	-1.3897781
C	-1.07222381	-12.8663087	-5.25063982	C	5.16595142	-12.5591858	0.83819849
C	-3.06846527	-13.4029132	-3.95086116	C	4.28983661	-13.6036796	-1.18735985
H	3.05451405	-10.1505831	5.62604788	H	-6.26308008	-9.11546494	1.59144383
H	4.383938	-11.2844914	5.99128988	H	-6.96581747	-9.9064294	3.02914505
H	4.51993316	-10.153322	4.60732253	H	-5.62038791	-8.73596289	3.21257386
H	1.83252901	-13.4173345	4.40342089	H	-4.88341663	-12.5430485	1.51583489
H	2.82729693	-13.1720907	5.87381259	H	-6.53922102	-12.1091216	2.04668732
H	1.48220004	-12.0580001	5.50553036	H	-5.83180047	-11.3408873	0.59912758
H	3.92293581	-13.4779882	3.149001	H	-4.15992636	-12.0619927	3.79482279
H	5.06198483	-12.095465	3.23633377	H	-4.47447559	-10.4496404	4.5142086
H	4.81794961	-13.1474181	4.65443038	H	-5.82425831	-11.5712738	4.20206394
H	-2.4173337	-10.5611468	-5.68962943	H	5.95802914	-10.6596008	-0.91737883
H	-3.461238	-11.9509359	-6.09636496	H	6.52201656	-12.2604751	-1.47089955
H	-3.83053798	-10.9356499	-4.66598718	H	5.23748363	-11.3964987	-2.37444632
H	-0.47351884	-13.5136614	-4.6095313	H	4.39116023	-13.0452875	1.43146009
H	-1.51796343	-13.4412065	-6.0640272	H	6.0320305	-13.214333	0.73111804
H	-0.45373366	-12.0677394	-5.65559714	H	5.46368572	-11.6239554	1.30821983
H	-2.47927811	-14.0941837	-3.34928949	H	3.55320704	-14.1338316	-0.58468044
H	-3.90186169	-13.0022974	-3.37479859	H	3.91572913	-13.4361639	-2.19673204
H	-3.44576928	-13.9153502	-4.83602142	H	5.21447936	-14.1796828	-1.22887054

References

1. C. R. Cantor, P. R. Schimmel, in *Biophysical Chemistry*, Vol. 3, WH Freeman and Co., San Francisco.

2. M. Egli, W. Saenger, in *Principles of Nucleic Acid Structure*, Springer-Verlag, New York, **1980**.

3. J. D. McGhee, P. H. V. Hippel, *J Mol. Biol.* **1974**, *86*, 469-489.

4. J. L. Mergny, L. Lacroix, *Oligonucleotides* **2003**, *13*, 515-537.
